# Myeloablative hematopoietic stem cell transplantation improves survival but is not curative in a pre-clinical model of myelodysplastic syndrome

**DOI:** 10.1371/journal.pone.0185219

**Published:** 2017-09-27

**Authors:** Yang Jo Chung, Terry J. Fry, Peter D. Aplan

**Affiliations:** 1 Genetics Branch, Center for Cancer Research, National Cancer Institute, National Institutes of Health, Bethesda, Maryland, United States of America; 2 Pediatric Oncology Branch, Center for Cancer Research, National Cancer Institute, National Institutes of Health, Bethesda, Maryland, United States of America; University of Kentucky, UNITED STATES

## Abstract

Allogeneic hematopoietic stem cell transplantation (A-HSCT) remains the only curative option for patients with myelodysplastic syndrome (MDS). We used the NUP98-HOXD13 (NHD13) murine model for MDS to study HSCT in a pre-clinical setting. NHD13 recipients transplanted with syngeneic bone marrow (S-HSCT) following myeloablative irradiation showed disease remission, with normalization of peripheral blood parameters and marked decrease in circulating leukocytes derived from the MDS clone. Despite the disease remission and improved survival compared to non-transplanted NHD13 controls, all mice eventually relapsed, indicating persistence of a long-lived radio-resistant MDS clone. In an effort to induce a graft versus leukemia (GVL) effect, A-HSCT with donor bone marrow that was mismatched at minor histocompatibility loci was compared to S-HSCT. Although recipients in the A-HSCT showed a lower early relapse rate than in S-HSCT, all mice in both groups eventually relapsed and died by 54 weeks post-transplant. To obtain a more significant GVL effect, donor splenocytes containing reactive T-cells were transplanted with allogeneic bone marrow. Although the relapse rate was only 20% at post-transplantation week 38, suggesting a GVL effect, this was accompanied by a severe graft versus host disease (GVHD) Taken together, these findings indicate that a myeloablative dose of ionizing radiation is insufficient to eradicate the MDS initiating cell, and that transplantation of donor splenocytes leads to decreased relapse rates, at the cost of severe GVHD. We suggest that NHD13 mice represent a feasible pre-clinical model for the study of HSCT for MDS.

## Introduction

The myelodysplastic syndromes (MDS) represent a heterogeneous group of clonal hematopoietic stem cell disorders with significant morbidity and high mortality [1–3]. The incidence of the disease is closely associated with age and increases dramatically after the age of 65. More than 10,000 new MDS cases are estimated to occur annually in the United States [4].

Although three agents (5-azacytidine, decitabine, and lenalidomide) have recently been FDA (U.S. Food and Drug Administration) approved for the treatment of MDS, and countless other drugs have been used off-label, the only curative option for patients with MDS is allogeneic hematopoietic stem cell transplantation (A-HSCT) [5]. However, only a minority of MDS patients are eligible for A-HSCT, due to lack of an appropriate donor, advanced age, or patient co-morbidities [6–9]. Mortality following A-HSCT for treatment of MDS can be separated into three general categories, MDS relapse, non-relapse mortality (NRM) and graft-versus-host disease (GVHD) [10, 11]. Compared to younger patients, older (age>60 yrs) individuals have higher rates of NRM and severe GVHD than younger individuals.

GVHD is often accompanied by a beneficial graft versus tumor (GVT) or graft versus leukemia (GVL) effect [12–14], especially in the context of myeloid malignancies[15, 16]. Several variables are thought to influence the overall event free survival following A-HSCT for MDS. These variables include conditioning regimens, HSC donor, HSC source (bone marrow or mobilized peripheral blood HSC), degree of GVHD/GVL, in addition to critical host variables, such as biologic and genetic features of the MDS clone [16–21]. Given the heterogeneity of these clinical variables, we propose using NUP98-HOXD13 (NHD13) transgenic mice [22] as a pre-clinical animal model to study the efficacy of A-HSCT. The NUP98-HOXD13 fusion was initially identified as a fusion gene produced by a t(2;11)(q31;p15.5) chromosomal translocation in patients with MDS [23, 24]. Mice that express an NHD13 transgene in the hematopoietic compartment develop a highly penetrant MDS that recapitulates the human disease in terms of peripheral blood cytopenias, ineffective hematopoiesis, dysplasia, and transformation to AML [22, 25]. We have previously shown that MDS generated by the NHD13 transgene can be transplanted in a cell-autonomous fashion, since transplantation of NHD13 bone marrow nucleated cells (BMNC) into syngeneic, wild-type (WT) recipients, transmits the disease as the NHD13 hematopoietic cells gradually outcompete host hematopoietic cells [25]. In this study, we evaluate the utility of NHD13 MDS mice as a pre-clinical animal model for MDS HSCT in terms of conditioning regimen, HSC donor, and GVL effect.

## Materials and methods

### Recipient mice with MDS

This animal work was approved by the NCI Animal Care and Use Committee, Protocol number GB-006. NHD13 mice, which were generated on a C57BL/6 background and express the CD45.2 isoform of CD45, are an established mouse model of MDS [22, 25]. NHD13 mice 4–10 months of age were used as transplant recipients. Complete blood counts (CBC) from NHD13 and wild-type (WT) mice of similar age were performed using a HEMAVET Multispecies Hematology Analyzer (CDC Technologies, Oxford, CT). The NHD13 recipients showed peripheral blood cytopenias consistent with MDS; any NHD13 candidate recipients that showed hematologic indices consistent with transformation to acute leukemia were excluded from transplantation experiments.

Humane endpoints were used for recipient mice. Mice showing clinical signs of leukemia, severe MDS, or GVHD, including hunched posture, lethargy, respiratory distress, inability to reach food, or masses >1 cm in any dimension were euthanized that day. Most mice in the study were euthanized when disease became apparent, but a minority were found dead in the cage. Euthanasia was performed using CO_2_ inhalation from a bottle source. These mice are detailed in Tables 1 and 2, S4 and S5 Tables.

**Table 1:** Clinical outcome of therapeutic HSCT in NHD13 mice with MDS.

Type of HSCT	Recipient ID	Follow-up (*weeks)	CBC Acquisition (*week)	WBC (K/uL)	ANC (K/uL)	Lym (K/uL)	HGB (g/dL)	MCV (fL)	Cause of Death	Diagnosis
S-HSCT	**#81**	26	26	41.52	30.29	5.02	3.1	68.8	**Relapse**	**AML**
	**#84**	42	40	7.4	2.65	4.35	13.3	43.5	**Relapse**	**MDS**
	**#90**	36	26	9.5	3.51	5.37	11.6	46.9	unknown	Non-relapse mortality
	**#169**	8	6	10.0	5.83	3.41	11.7	48.8	**Relapse**	**MDS**
	**#177**	46	46	3.84	1.25	1.89	9.6	47.9	**Relapse**	**MDS**
	**#178**	39	36	3.02	1.45	1.13	6.6	53.9	**Relapse**	**MDS**
	**#179**	37	37	96.06	24.76	60.01	5.8	55.2	**Relapse**	**T-ALL**
A-HSCT	**#238**	36	36	10.08	5.03	3.26	9.4	75.6	**Relapse**	**Erythroleukemia**
	**#231**	50	50	10.9	0.01	8.36	3.0	53.5	**Relapse**	**T-ALL** (pleural effusion)
	**#230**	38	38	6.96	3.91	2.66	11.9	63.6	**Relapse**	**T-ALL** (Thymoma)
	**#237**	39	39	1.51	0.77	0.63	15.1	44.7	unknown	Non-relapse mortality (seizure)
	**#307**	23	16	3.74	0.41	2.91	11.8	49.8	**Relapse**	Leukemia^a^
	**#309**	26	26	43.7	0.44	5.07	5.0	34.5	**Relapse**	**AML**
	**#312**	24	24	141.9	18.21	112.3	4.3	51.9	**Relapse**	**T-ALL**
	**#316**	54	54	15.02	3.0	10.14	4.2	53.5	**Relapse**	**MDS**
	**#324**	27	24	2.68	0.6	1.78	8.2	59.1	**Relapse**	**MDS**
	**#343**	12	12	239.7	76.8	124.7	8.5	90.8	**Relapse**	**AML**
	**#355**	27	24	6.6	1.27	4.63	10.9	41.9	**Relapse**	**MDS**
	**#334**	10	6	8.5	1.59	6.31	12.9	45.1	**Relapse**	Leukemia^a^

* Week after transplantation

^a^ “Leukemia” indicates leukemia not otherwise specified (NOS) for mice found dead with hepatosplenomegaly at necropsy

**Table 2:** Clinical outcome of A-HSCT with induced GVHD/GVL.

Type of HSCT	Recipient ID	Follow-up (*weeks)	CBC Acquisition (*week)	WBC (K/uL)	ANC (K/uL)	Lym (K/uL)	HGB (g/dL)	MCV (fL)	Cause of Death	Diagnosis
A-HSCT with splenocytes	#205	8	8	16.0	12.8	2.47	14.7	56.5	**GVHD**	**GVHD**
	#236	41	41	2.29	0.01	1.14	9.5	43.0	**GVHD**	**GVHD**
	#235	42	38	8.8	4.0	4.45	15.2	53.9	unknown	Non-relapse mortality
	#228	25	24	68.8	22.35	36.02	5.0	53.3	**Relapse**	**AML**
	#227	23	16	16.26	6.64	0.33	11.5	59.5	**GVHD**	**GVHD**

* Week after transplantation;

A total of 51 NHD13 mice were used as recipients in this study. All recipient mice were monitored by certified veterinarian technicians daily and research staff at least bi-weekly, or more often when clinically indicated, for up to 15 months post-transplantation. No surgical procedures were performed on the recipient mice post-transplantation, and no analgesics or anesthetics were given to the recipients. All research staff had taken the course “Using Animals in NIH Intramural Research”.

### Donor mice

For syngeneic hematopoietic stem cell transplants (S-HSCT), we used congenic mice, B6LY5.1/Cr (CD45.1) as donors. The mice were purchased from Charles River and maintained under micro-isolation conditions. For allogeneic hematopoietic stem cell transplantation (A-HSCT), C3H.SW mice, which are identical to C57BL/6 mice at the major histocompatibility loci, but have numerous mismatches at minor histocompatibility loci[26, 27], were used as the donor. The age range of donor mice was 4–8 months for all transplantation experiments. A total of 10 mice were used as donors in this study.

### Transplantation procedure and engraftment evaluation

Bone marrow nucleated cells (BMNC) were obtained from the femur and tibia of donor mice and used as bulk dissociated bone marrow without RBC lysis or further processing. 1X10^6^ BMNC per mouse were injected into NHD13 recipient mice following irradiation with 10 Gy (myeloablative) or 6.5 Gy (non-myeloablative) for S-HSCT[28]. For A-HSCT, 1X10^7^ BMNC (non-T cell depleted) were injected into NHD13 recipient mice following myeloablative irradiation; the higher cell dose was used to increase the probability of both engraftment and GVHD/GVL in the mismatched donor setting, as previously reported [29]. All the cell injections were done via tail vein using a 30 g needle. In some experiments, CD4+CD25+ T-reg cells were isolated from donor splenocytes using CD4+CD25+ Regulatory T Cell (Treg) Isolation Kit (Miltenyi Biotec, Gladbach, Germany). 4 to 5 X 10^5^ T-reg cells were injected into recipients along with 1X10^7^ BMNC following myeloablative irradiation. The Treg cell dose was based on a previous report[30]. Ciprofloxacin water (100mg/L) was provided to recipients for three weeks after the transplantation.

Donor lymphocyte infusion (DLI) was performed beginning at post-transplantation week 12, followed by monthly re-infusions until the detection of disease relapse in the recipients. The cell dose used for DLI experiments was 1.8X10^7^ donor splenocytes per recipient, which contained approximately 3X10^6^ CD3 T cells[19]. For experiments intended to induce GVHD at the time of transplant, 1X10^7^ freshly isolated donor splenocytes containing 1.7X10^6^ CD3 T cells were mixed with 1X10^7^ donor BMNC and injected into recipient mice following myeloablative irradiation. The CD3 T cells numbers were based on flow cytometry analysis of splenocytes from a representative healthy C3H.SW mouse after staining donor splenocytes with anti CD3 antibody.

Engraftment assays were performed with peripheral blood (PB) obtained from the tail vein using di-potassium EDTA (ethylenediaminetetraacetic acid) salt as an anticoagulant. Each PB sample was divided into samples for flow cytometry and CBC, analyzed as described above.

### Monitoring GVHD and evidence of relapse

Recipient mice were observed daily, and GVHD was assessed using five clinical parameters: weight loss, posture, activity, fur texture and skin integrity. Individual mice received a score of 0 to 2 for each criterial (maximum score of 10) [31, 32]. CBCs were assessed at 1–3 month intervals, and mice were observed for signs of disease (such as hunched posture, lethargy, non-responsiveness).

### Flow cytometry

To evaluate donor chimerism, PB samples were treated with a red blood cell (RBC) lysis buffer, and stained with CD45.2 (Ly5.2)-Cy5 (eBioscience) for S-HSCT or Ly9.1-FITC (BD Pharmingen) for A-HSCT, as follows. The cells were resuspended with Hank’s Balanced Salt Solution (HBSS) (Ca^2+^, Mg^2+^ free, Invitrogen, CA) containing 2% fetal bovine serum (FBS) (HF2 solution) and then incubated for 30 min on ice with fluorochrome-conjugated antibodies. Following staining, cells were washed twice with phosphate buffered saline (PBS) and then resuspended with HF2 containing 1ug/ml of propidium iodide (Sigma). The analyses were performed using a dual laser FACScan (BD Bioscience). Mice that were clinically ill were euthanized using CO_2_ narcosis. BM, spleen, and thymus were harvested and stained with antibodies to Mac-1-PE (BD Pharmingen), Gr-1-FITC (BD Pharmingen), B220-APC (BD Pharmingen), CD4-PE (eBioscience), CD8-FITC (eBioscience), CD71-PE (eBioscience), Ter119-FITC (BD Pharmingen), cKit-FITC (BD Pharmingen), Sca-1-PE (BD Pharmingen), and analyzed as described above. Cell quest pro version 5.2.1 (BD Bioscience) software was used for flow cytometry analysis.

### Statistics

Statistical evaluation was done by using Student’s t test and Excel (Microsoft, Redmond WA) software and Log-Rank test with GraphPad Prism (La Jolla, CA) software.

## Results

### A myeloablative conditioning regimen can induce remission in NHD13 mice with MDS

**Fig 1:**
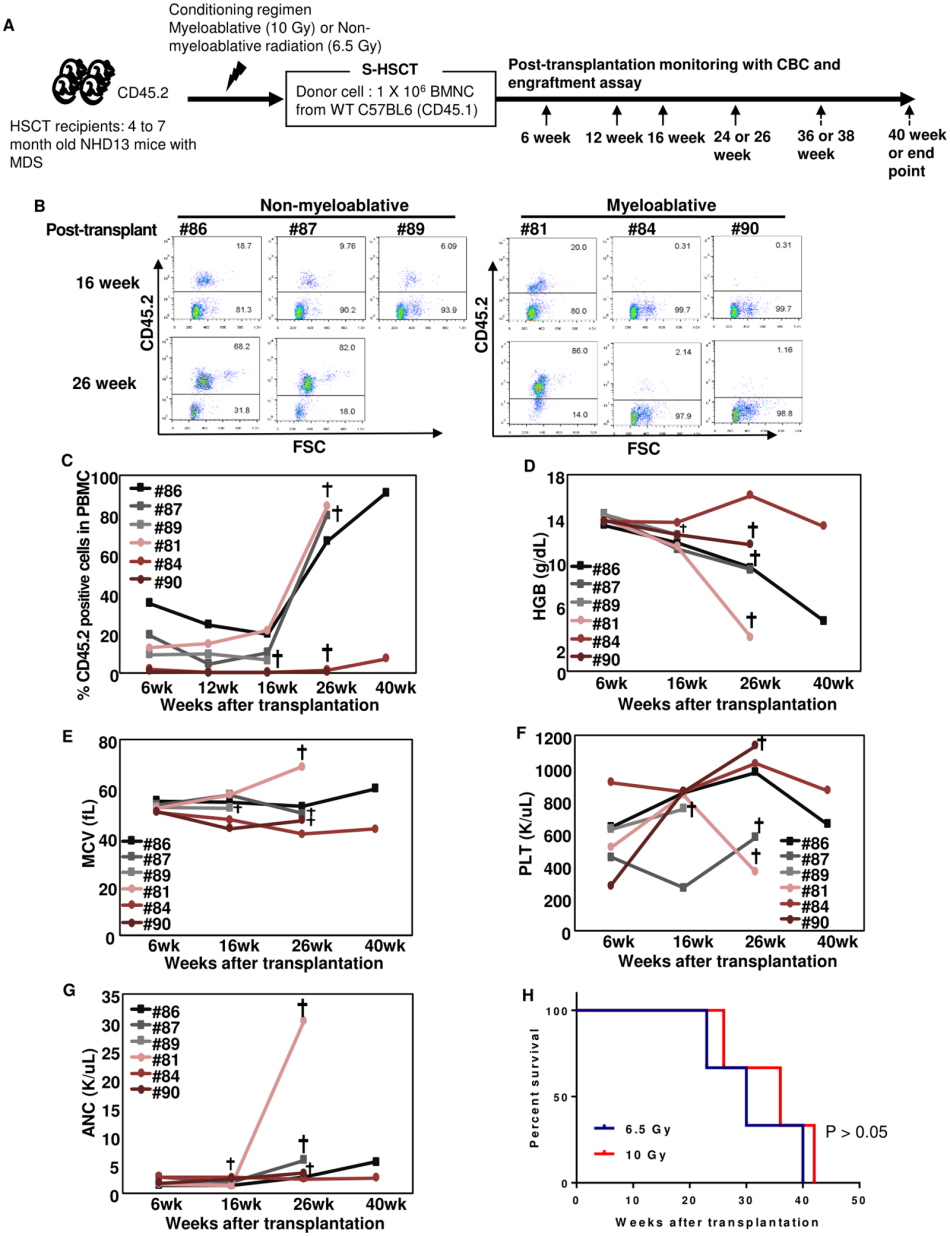
Syngeneic hematopoietic stem cell transplant (S-HSCT) as therapy for MDS. (A) Schematic illustration of S-HSCT experiment, using myeloablative (10 Gy) or non-myeloablative (6.5 Gy) total body irradiation as a conditioning regimen (B) Representative FACS profiles of individual recipients at 16 and 26 weeks post-transplant. CD45.2+ indicates cells derived from the host. (C) CD45.2+ cells from individual recipients; increasing CD45.2+ cells indicate relapse of host (MDS-derived) hematopoiesis. (D) Hemoglobin changes following S-HSCT. (E) MCV, Mean corpuscular volume. (F) PLT, platelet count. (G) ANC, absolute neutrophil count. (H) Survival curve after HSCT. The sword symbol indicates death of the recipient.

We used the NUP98-HOXD13 (NHD13) transgenic model of MDS, which we and others have shown is highly penetrant and recapitulates the key features of human MDS, including peripheral blood cytopenias, dysplasia, ineffective hematopoiesis, and transformation to AML [22, 33]. To determine an irradiation dose that would ablate host MDS cells, we evaluated cesium source radiation doses of 6.5 Gy (non-myeloablative) or 10 Gy (myeloablative). NHD13 mice aged 4 to 10 months of age with evidence of MDS (see S1 and S2 Tables for details) were transplanted with WT bone marrow nucleated cells (BMNC), obtained from congenic C57BL/6 donors 4–8 months of age (Fig 1A). Since the transplant donor and recipients were both of C57BL/6 origin, we referred to these transplants as syngeneic HSCT (S-HSCT). The NHD13 recipients were positive for the CD45.2 allele of CD45, while the WT donor was positive for the CD45.1 allele, thus allowing discrimination between host and donor hematopoiesis by flow cytometry.

None of the mice that received non-myeloablative radiation eliminated the MDS clone, since the peripheral blood showed >5% CD45.2 cells derived from the MDS clone at all time points assessed (Fig 1B and 1C). Furthermore, these mice showed progressive anemia and macrocytosis, with no mice surviving beyond 40 weeks post-transplant (Fig 1D–1H). In contrast, two of three NHD13 recipients (#84 and #90) that received myeloablative radiation showed normal peripheral blood indices, derived from donor (CD45.1) hematopoietic cells, for more than 26 weeks after HSCT (Fig 1B–1G; S3 Table), indicating that they were in a continuous remission. These findings indicated that myeloablative radiation (10 Gy) could produce a sustained hematologic remission in mice with MDS, whereas non-myeloablative irradiation (6.5 Gy) could not. Therefore, subsequent experiments with this model used myeloablative radiation as a conditioning regimen. Despite the presence of <2% CD45.2 cells, and normal hematopoietic indices at 26 weeks post-transplant, all mice treated with myeloablative radiation eventually relapsed (with MDS) by 42 weeks post-transplant, indicating that although a lethal, myeloablative dose of radiation could lead to long term remission, it was not curative in this MDS model, due to late relapse of disease. A second independent experiment yielded similar results (S1 Fig). These late relapses suggest the persistence of a long-lived, radio-resistant MDS initiating cell.

### Allogeneic versus syngeneic HSCT as treatment for MDS

**Fig 2:**
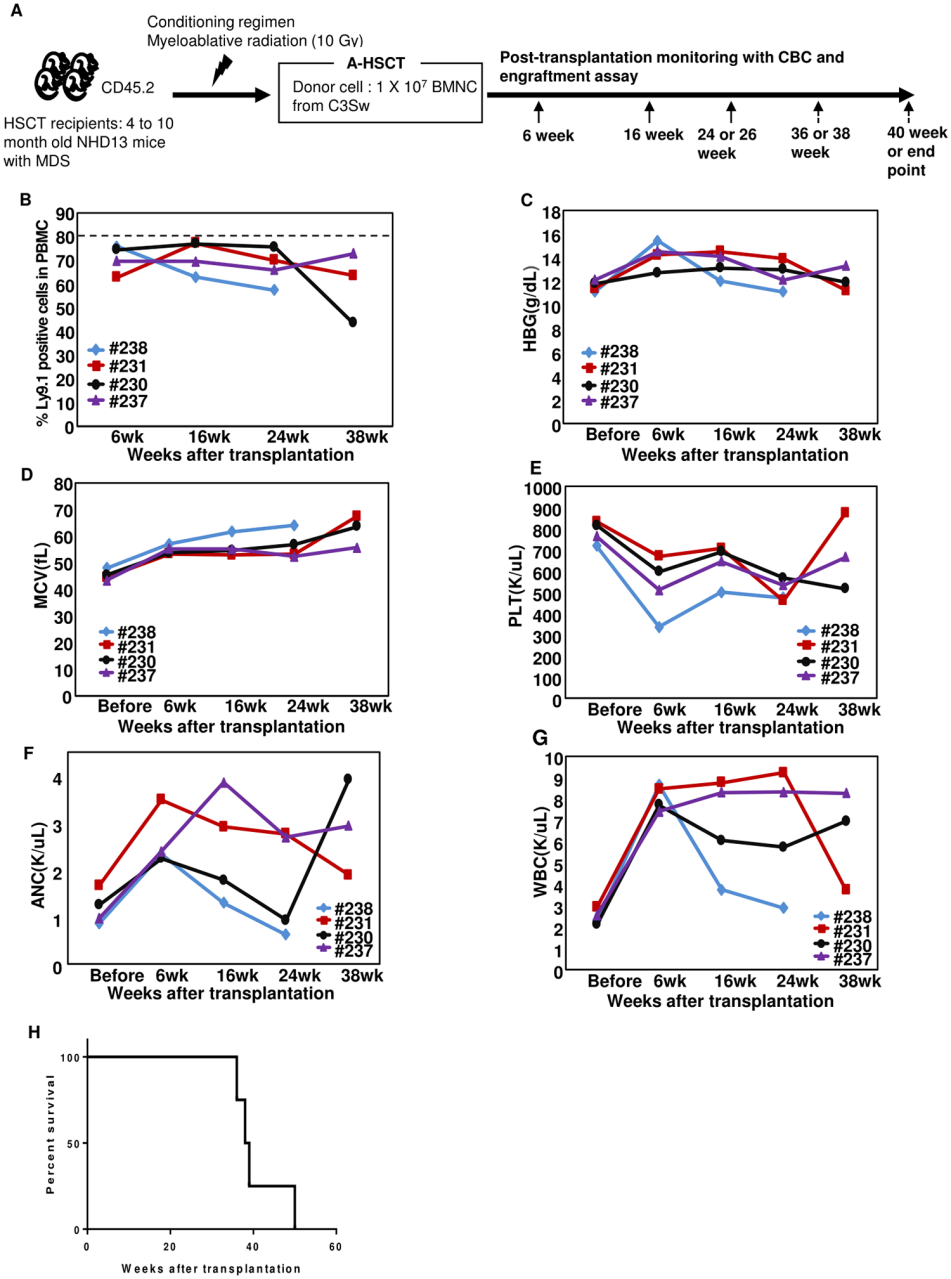
Evaluation of allogeneic HSCT (A-HSCT) using the NHD13 model of MDS. (A) Schematic illustration of AHSCT experiment. (B) Engraftment of donor cells was evaluated using an anti-Ly9.1 antibody. The dotted line indicates Ly9.1 positivity (79.9 ± 2.5%) in the peripheral blood of 5 healthy C3H.sw donor mice, as a reference for full donor chimerism. (C) HGB, hemoglobin. (D) MCV, Mean corpuscular volume. (E) PLT, platelet count. (F) ANC, absolute neutrophil count. (G) WBC, white blood cell count. (H) Survival curve for this cohort only (n = 4). Data presented here is representative of three independent experiments; complete data for all experiments is presented in S3 Fig.

Allogeneic HSCT (A-HSCT) are effective treatments for myeloid malignancies due to a combination of profound cytotoxicity caused by a myeloablative conditioning regimen, as well as an immunological graft versus leukemia (GVL) effect. To evaluate the ability of A-HSCT to generate GVL, and its impact on long term survival post-transplant, we transplanted NHD13 mice that showed evidence of MDS, but had not transformed to AML, with allogeneic donor cells. The donor cells were obtained from C3H.Sw mouse bone marrow. C3H.Sw mice are matched with NHD13 mice (C57BL/6 background) at the major histocompatibility locus (MHC), but have numerous mismatches at minor histocompatibility loci[26]. This strategy (Fig 2A) has been used successfully to study graft versus host disease (GVHD) in mice[26, 27].

Since both C3H.Sw and the NHD13 mice expressed the CD45.2 isoform of CD45, we could not use CD45.2 to distinguish donor from recipient cells. Therefore, we used the Ly9.1 antigen, which is expressed on C3H.Sw lymphocytes and monocytes, but not expressed on C57BL/6 hematopoietic cells, as previously reported[34]. Recipient mice were irradiated with myeloablative radiation, followed by transplantation with 1 x 10^7^ BMNC from a healthy C3H.Sw donor. All mice engrafted, as evidenced by 60–90% donor Ly9.1 positive cells in the peripheral blood (Fig 2B). CBCs normalized, and the mice did well for >24 weeks (Fig 2C–2G). No mice developed clinical signs of GVHD such as fur loss, hunched posture, or weight loss. However, as early as 16 weeks post-transplant one animal (#238) began to relapse, as evidenced by decreased Ly9.1 cells in the peripheral blood, accompanied by decreased HGB, ANC, WBC, and increased MCV. Eventually, three (#231, #230 and #237) of four mice developed leukemia between 36–50 weeks, and the fourth died of unknown causes after a seizure (Fig 2H). Curiously, two (#231 and #230) of the three fatal leukemias that developed were of T-cell origin (Table 1 and S2 Fig).

**Fig 3:**
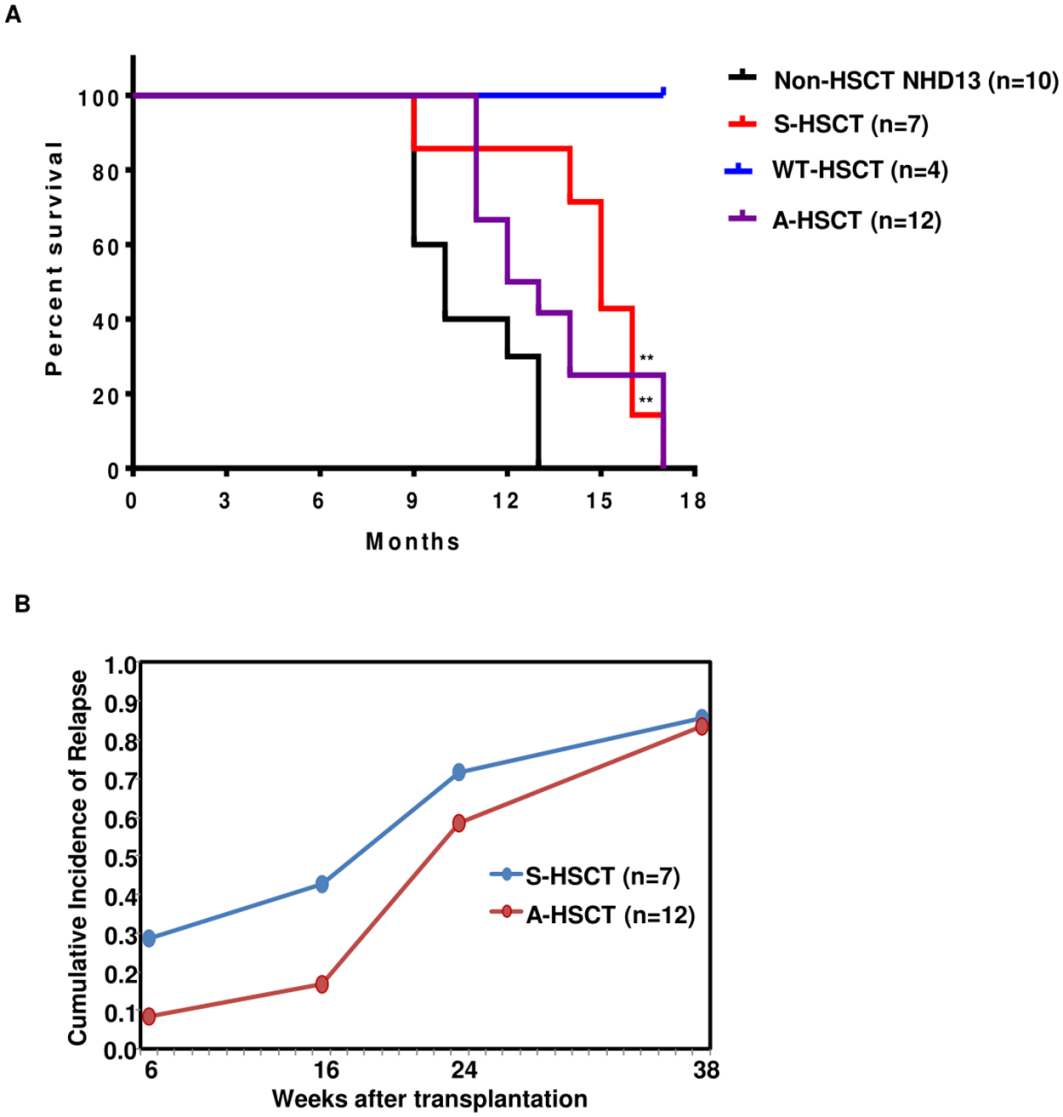
Overall survival and cumulative incidence of relapse. (A) Log RANK test for Non-HSCT vs S-HSCT, p = 0.0013; Non-HSCT vs A- HSCT, p = 0.0054; Allo HSCT vs Syn HSCT, p = 0.7373. Non-HSCT NHD13 refers to NHD13 transgenic mice not subjected to myeloablative irradiation and HSCT. WT-HSCT refers to WT recipient mice transplanted with WT syngeneic BMNC following myeloablative irradiation. (B) Cumulative incidence of relapse for S-HSCT and A-HSCT.

The A-HSCT experiments was repeated two additional times, using different C3H.Sw donors, to determine if the results were reproducible (S3 Fig). Fig 3A shows that both syngeneic and allogeneic transplants led to a survival advantage compared to non-transplanted NHD13 mice, with a median survival of 15 months in S-HSCT and 13.5 months in A-HSCT compared with non-transplanted NHD13 mice (median survival 10 months) (details shown in Table 1). Careful examination of serial CBCs and engraftment (Figs 1 and 2) indicated that several mice (#84, #231, #230 and #237) from both the S-HSCT and A-HSCT groups had prolonged (> 26 weeks) remissions prior to relapse, suggesting that relapse was due to persistence of a quiescent long-lived MDS stem/initiating cell. The onset of relapse was somewhat delayed in the mice receiving A-HSCT as compared to S-HSCT (Fig 3B), suggesting the potential for a graft versus leukemia (GVL) effect. We used donor lymphocyte infusion (DLI) following A-HSCT in an attempt to increase a GVL effect. 3 X 10^6^ donor lymphocytes (freshly obtained from spleen of C3H.Sw) were infused via tail vein injection at 13 weeks post-A-HSCT, and monthly thereafter. However, there were no signs of GVHD in these mice, and we noted no difference in survival, relapse (as evidenced by increasing numbers of host cells), HGB, ANC, or platelet count in the DLI group compared to A-HSCT with no DLI (S4 Fig; S4 Table).

### Graft versus host disease (GVHD) and relapse

**Fig 4:**
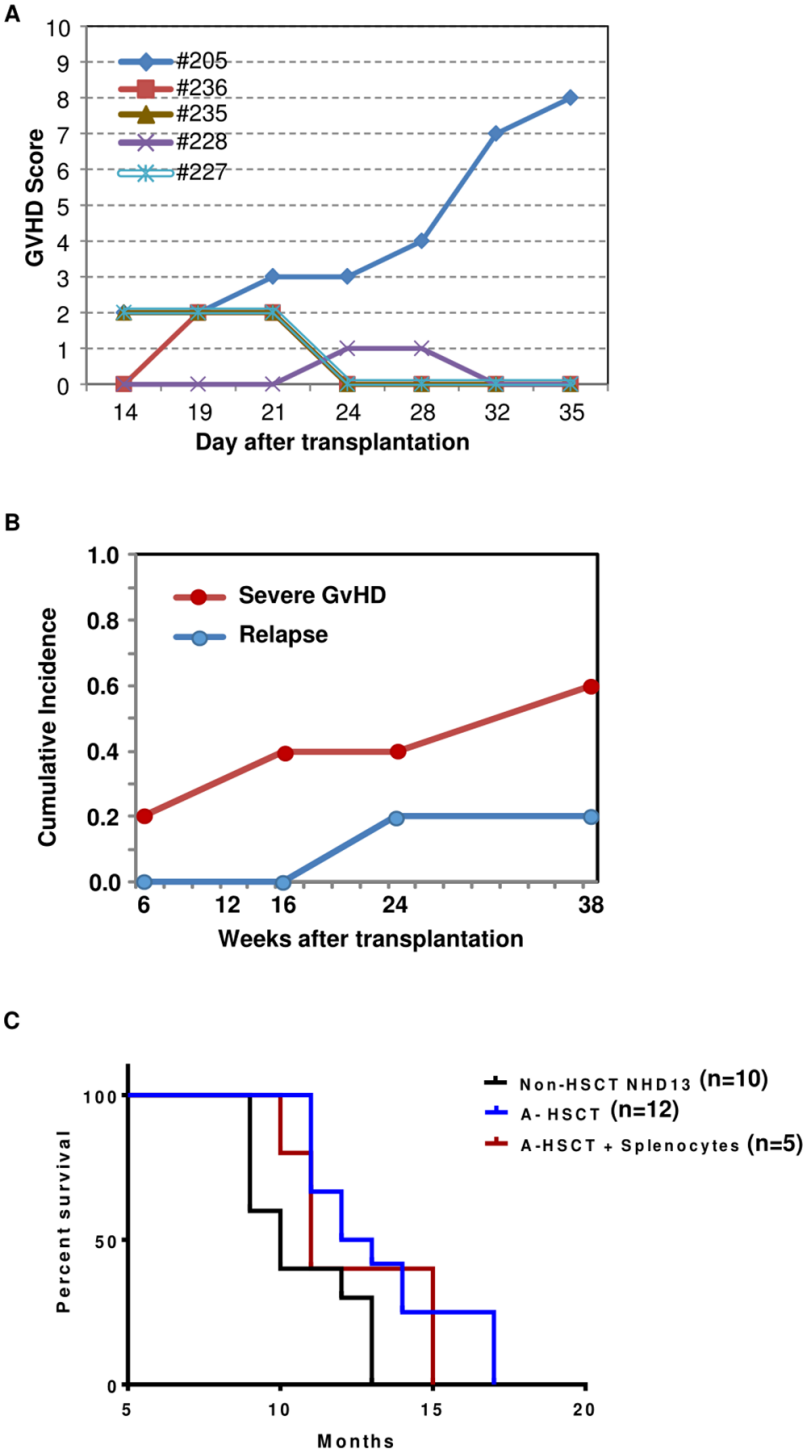
Effect of GVHD using the NHD13 A-HSCT model. (A) GVHD was induced by transplantation of splenocytes with BMNC. GVHD score vs time for individual recipients; the values for #227 and #235 are identical and thus the curves are superimposed on one another(B) Cumulative incidence of severe GVHD or relapse. Mice were euthanized when they displayed clinical signs of disease such as lethargy, tachypnea, or hunched posture. (C) Survival curve comparison of Non-HSCT NHD13, A-HSCT and A-HSCT + Splenocytes. Non-HSCT NHD13 refers to NHD13 transgenic mice not subjected to irradiation and HSCT.

Low grade or chronic GVHD is a favorable prognostic feature in patients with MDS, presumably due to a Graft versus leukemia (GVL) effect that accompanies the GVHD [35, 36]. Because the A-HSCT recipients that received unfractionated donor BMNC did not show signs of GVHD, we added 1 x 10^7^ splenocytes (containing approximately 1.7 x 10^6^ CD3+ T-lymphocytes) of donor origin to 1 x 10^7^ donor BMNC at the time of transplant to induce GVHD and GVL. Four (#205, #236, #235 and #227) of five recipients showed a GVHD score of at least two at post-transplantation day 19 (Fig 4A; S5 Fig). The recipient with the highest GVHD score (#205) showed no signs of MDS with a normal CBC, but was euthanized 8 weeks post-transplant due to severe GVHD (S5 and S6 Figs). Two mice with acute GVHD scores ≥2 were long term survivors (#239 and #236), with no evidence for MDS at 38 weeks post transplant based on engraftment level and CBC. However, one of these two (#236) was euthanized at 41 weeks post-transplant due to lethargy, hunched posture, and hair loss, consistent with chronic GVHD; this mouse had no signs of MDS at necropsy (S6 Fig). A second long-term survivor (#235) had a normal CBC, and high level engraftment of Ly9.1 cells, but died of unknown causes at 42 weeks post-transplant (Table 2). Recipient #228 was the only recipient to show no signs of GVHD at transplant day 19 and relapsed with AML at 24 weeks post-transplant (Table 2). Fig 4C shows that transplant conditions which induced GVHD (A-HSCT + splenocytes) led to a survival advantage compared to non-transplanted NHD13 mice, but no survival advantage compared to A-HSCT recipients, largely due to severe GVHD. These results suggested a correlation between GVHD and prolonged remission; in fact, the primary cause of death in this group was not disease relapse, but was instead GVHD (Fig 4B and 4C; S5 Fig).

### Transplantation of Treg cells did not improve GVL effect

Given a suggestion from the literature [30] that Treg cells could confer a GVL effect in the absence of GVHD, we transplanted mice with 4–5 x 10^5^ C3H.Sw Treg cells in addition to 1 x 10^7^ C3H.Sw BMNC. Serial samples were analyzed for hematologic indices and engraftment. Similar to prior results with A-HSCT, several mice showed normal hematologic parameters and no evidence of relapse for up to 24 weeks (S7 Fig; S5 Table). However, all mice eventually died of relapsed MDS or leukemia, excepting one mouse that died of unknown causes at 10 weeks post-transplant. Comparison of A-HSCT with Treg cells demonstrated a survival benefit compared to non-transplanted NHD13 mice with MDS but did not improve survival compared to A-HSCT alone (S7 Fig).

## Discussion

Allogenic HSCT remains the only curative therapy for individuals with MDS; however, many aspects of this successful treatment approach remain unclear due to the heterogenous nature of the treatment (conditioning regimen, source of HSC, immunosuppressive agents) and the heterogeneity of the disease (genetic factors, prior treatments). Pre-clinical animal models allow one to control for some of these variables. For instance, the NHD13 mice with MDS all have a common inciting event (the NHD13 transgene), and have no prior treatment history, in contrast to patients with MDS who may come to transplant after receiving numerous genotoxic therapies. Alternate donor sources can be studied using recipients that have a common genetic lesion. Given the shorter life-span of mice, it becomes practical to follow the recipients for the remainder of their natural lives.

Total body irradiation, alone or in combination with myeloablative agents such as busulfan or cyclophosphamide, has been used as a conditioning regimen prior to HSCT for patients with MDS [17, 18]. Our initial experiments indicated that although myeloablative radiation could lead to short term remission in mice with MDS, all mice eventually relapsed. These results suggested that therapeutic failure was due to the persistence of a long-lived, malignant, radio-resistant clone.

Given that a GVL effect is thought to provide a survival benefit in HSCT studies of MDS patients, we attempted to provide a therapeutic GVL effect in this model, using donors that were matched at the major MHC, but mismatched at minor histompatibility loci. Our initial studies showed minimal GVHD, but no survival effect. A first attempt to increase GVHD was via donor lymphocyte infusions; this approach also failed to induce significant GVHD or survival benefit. A third approach was to infuse mismatched donor T cells at the time of transplant; this therapy induced GVHD, but no survival benefit over syngeneic donors, where the therapeutic benefit is provided by myeloablative conditioning only. Finally, the use of Treg cells to produce a selective GVL effect resulted in little GVHD, but no evidence for a survival benefit due to GVL effect either.

Given recent reports [37, 38] that showed efficacy of Tregs in inducing a selective GVL effect in both a clinical and xenotransplant model, we were disappointed to see no benefit of Tregs in our model. However, there were several differences between our study and the recently reported pre-clinical study [37], including the disease treated (MDS vs xeno-transplanted acute leukemia), Treg dosage (4 x10^5^ vs 3 x 10^6^), and source of Tregs (minor mismatched mice vs. random human donors), which may help to explain this apparent discrepancy. Of note, there was a trend toward later relapse in recipients of mismatched HSC (Fig 3B), although overall survival was not significantly different, and mice with severe GVHD had a decreased incidence of relapse (Fig 4B), again without an improved overall survival. Taken together, these findings suggest that a GVL effect can be achieved in this model, but that the toxic effects of severe GVHD need to be better controlled if a survival benefit is to be achieved.

The role of aberrant stroma, and aberrant microenvironment in MDS has been a topic of intense interest [39–43]. In one study, murine bone marrow stromal cells deficient in Dicer induced MDS and AML in WT hematopoietic cells [40]. In a second study, human bone marrow stromal cells derived from MDS patients supported the growth of human MDS hematopoietic cells in xeno-transplant assays using immunodeficient mice, whereas bone marrow stromal cells derived from normal volunteers did not support growth in those xeno-transplant assays [42]. However, in the current study, all of the leukemias that developed in the NHD13 transplant recipients were of host (CD45.2) origin, suggesting that the NHD13 bone marrow stromal cells did not transform WT (CD45.1) donor cells.

In summary, having established the basic framework for a murine model of therapeutic HSCT for MDS, one can envision numerous avenues to improve this efficacy. For instance, myeloablative regimens more effective at targeting quiescent HSC, post-transplant therapy of mice in remission, aggressive treatment of GVHD, and use of alternative donors are just a few variables that can be manipulated. The inbred NHD13 mice can be outbred, and then transplanted with parental donor cells, mimicking haplo-identical transplants in humans.

## Supporting information

S1 Fig Syngeneic hematopoietic stem cell transplant (S-HSCT) as therapy for MDS. (A) Schematic illustration of S-HSCT experiment, using lethal TBI as a conditioning regimen. (B) CD45.2+ cells from individual recipients; increasing CD45.2+ cells indicate relapse of host (MDS-derived) hematopoiesis. (C) Hemoglobin changes following S-HSCT. (D) MCV, Mean corpuscular volume. (E) PLT, platelet count. (F) ANC, absolute neutrophil count. (G) WBC, white blood cell count. Line color (blue or black) indicates different experiment cohorts and symbols represent individual recipients. Data in this figure show the results of two independent HSCT experiments.
(TIF)

S2 Fig Diagnostic flow cytometry analysis of A-HSCT recipients at study end point. (A) Erythroleukemia, Note increased erythroblast (CD71+/Ter119+) cells in spleen and peripheral blood (PB). (B) and (C) T-ALL characterized by T cell infiltration of BM and predominance of host (Ly9.1 negative) cells. (D) this recipient showed normal immunophenotypic characteristics and predominance of donor cells in spleen. No sign of relapse.
(TIF)

S3 Fig Allogeneic hematopoietic stem cell transplant (A-HSCT) as therapy for MDS. (A) Schematic illustration of A-HSCT experiment. (B) Engraftment of donor cells were evaluated using an anti-Ly9.1 antibody. The dotted line indicates Ly9.1 positivity (79.9 ± 2.5%) in the peripheral blood of 5 healthy C3H.sw donor mice, as a reference for full donor chimerism. (C) HGB, hemoglobin. (D) MCV, Mean corpuscular volume. (E) PLT, platelet count. (F) ANC, absolute neutrophil count. (G) WBC, white blood cell count. (H) Survival curve (n = 12). Three independent A-HSCT experiment cohorts are represented with three different color lines (black, blue and green). Each symbol indicates individual HSCT recipients.
(TIF)

S4 Fig Evaluation of allogeneic HSCT (A-HSCT) with Donor Lymphocyte Infusion (DLI) using the NHD13 model of MDS. (A) Schematic illustration of the experiment. DLI started at post-transplant week 13 and donor lymphocytes were injected every 4 weeks until the endpoint of the experiment. (B) Engraftment of donor cells were evaluated using an anti-Ly9.1 antibody as described in S3 Fig. (C) HGB, hemoglobin. (D) MCV, Mean corpuscular volume. (E) PLT, platelet count. (F) ANC, absolute neutrophil count. (G) WBC, white blood cell count. (H) Comparing survival curves from A-HSCT and A-HSCT with DLI recipient group. Two independent A-HSCT experiment cohorts are represented by different color lines (black and blue).
(TIF)

S5 Fig A-HSCT with concurrent splenocytes to induce graft versus host disease (GVHD). Myeloablative recipient MDS mice were transplanted with allogeneic donor BM along with allogeneic donor splenocytes to induce GVHD. (A) Representative photos of recipients that developed GVHD, # indicates the recipient animal’s identification number. (B) Engraftment of donor cells were evaluated using an anti-Ly9.1 antibody as described in S3 Fig. (C) HGB, hemoglobin. (D) MCV, Mean corpuscular volume. (E) PLT, platelet count. (F) ANC, absolute neutrophil count. (G) WBC, white blood cell count.
(TIF)

S6 Fig Flow cytometry of mice with GVHD following A-HSCT plus splenocyte transplant at time of euthanasia show no evidence of malignancy. Two recipients that developed GVHD show high level of donor cell engraftment and normal FACS profiles in terms of lymphoid and myeloid cells in hematopoietic tissues.
(TIF)

S7 Fig Evaluation of allogeneic HSCT (A-HSCT) plus regulatory T cells (Treg) using the NHD13 model of MDS. (A) Schematic illustration of A-HSCT plus Treg. CD4+CD25+ T cells were isolated from donor spleen using magnetic cell sorting system (MACS). 4 to 5 X 10^5^ Treg cells were transplanted to lethally irradiated MDS recipients along with 1X10^7^ of whole BM cells. (B) Engraftment of donor cells were evaluated using an anti-Ly9.1 antibody as in S3 Fig. (C) HGB, hemoglobin. (D) MCV, Mean corpuscular volume. (E) PLT, platelet count. (F) ANC, absolute neutrophil count. (G) WBC, white blood cell count. (H) Comparison of survival curves from A-HSCT and A-HSCT with Treg.
(TIF)

S1 Table Complete Blood Counts (CBC) of C57BL/6 wild type mice. (DOC)

S2 Table CBC of NHD13 transgenic recipients before HSCT. (DOC)

S3 Table CBC following HSCT of MDS mice using Non-myeloablative (6.5 Gy) or Myeloablative (10 Gy)TBI as conditioning regimen. (DOC)

S4 Table Clinical outcome of allogeneic HSCT with donor lymphocyte infusion (DLI). (DOC)

S5 Table Clinical outcome of allogeneic HSCT with regulatory T cells (Treg). (DOC)
