# Supplementary figures and images for: Myeloablative hematopoietic stem cell transplantation improves survival but is not curative in a pre-clinical model of myelodysplastic syndrome
Source: PLoS One. 2017 Sep 27;12(9):e0185219. doi: 10.1371/journal.pone.0185219 (PMC5617185; doi:10.1371/journal.pone.0185219)

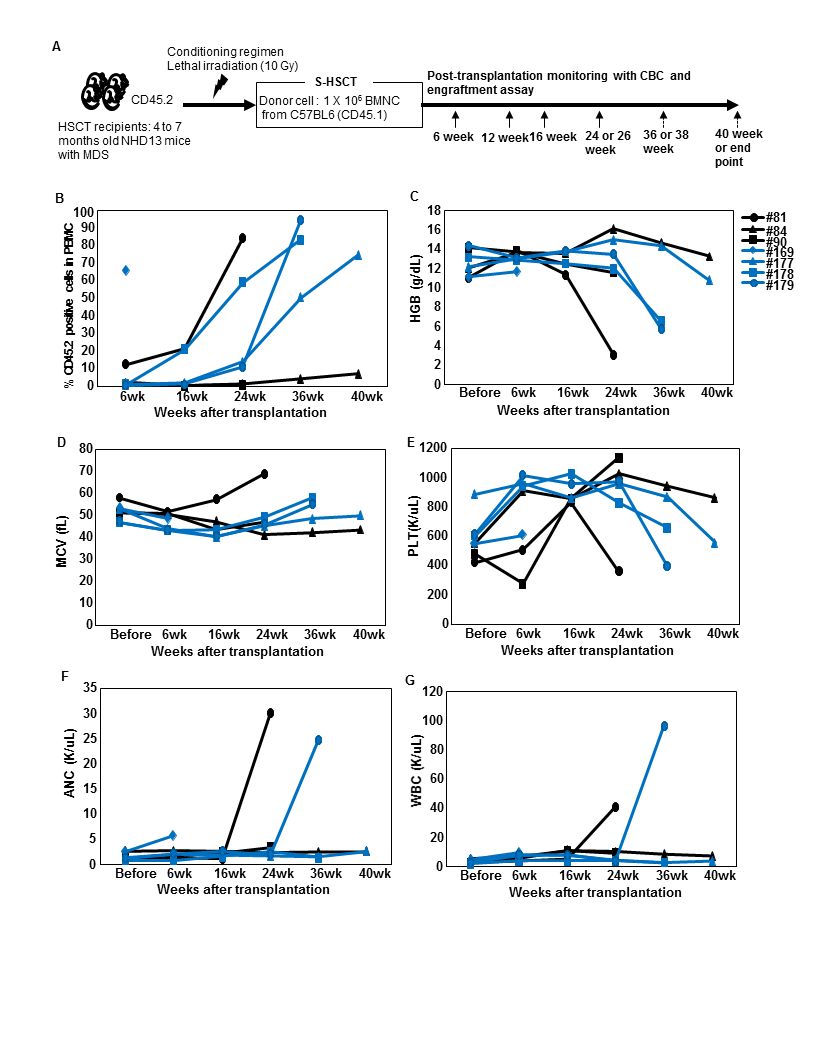

Supplement: S1 Fig — (A) Schematic illustration of S-HSCT experiment, using lethal TBI as a conditioning regimen. (B) CD45.2+ cells from individual recipients; increasing CD45.2+ cells indicate relapse of host (MDS-derived) hematopoiesis. (C) Hemoglobin changes following S-HSCT. (D) MCV, Mean corpuscular volume. (E) PLT, platelet count. (F) ANC, absolute neutrophil count. (G) WBC, white blood cell count. Line color (blue or black) indicates different experiment cohorts and symbols represent individual recipients. Data in this figure show the results of two independent HSCT experiments. (TIF) [file pone.0185219.s001.TIF]

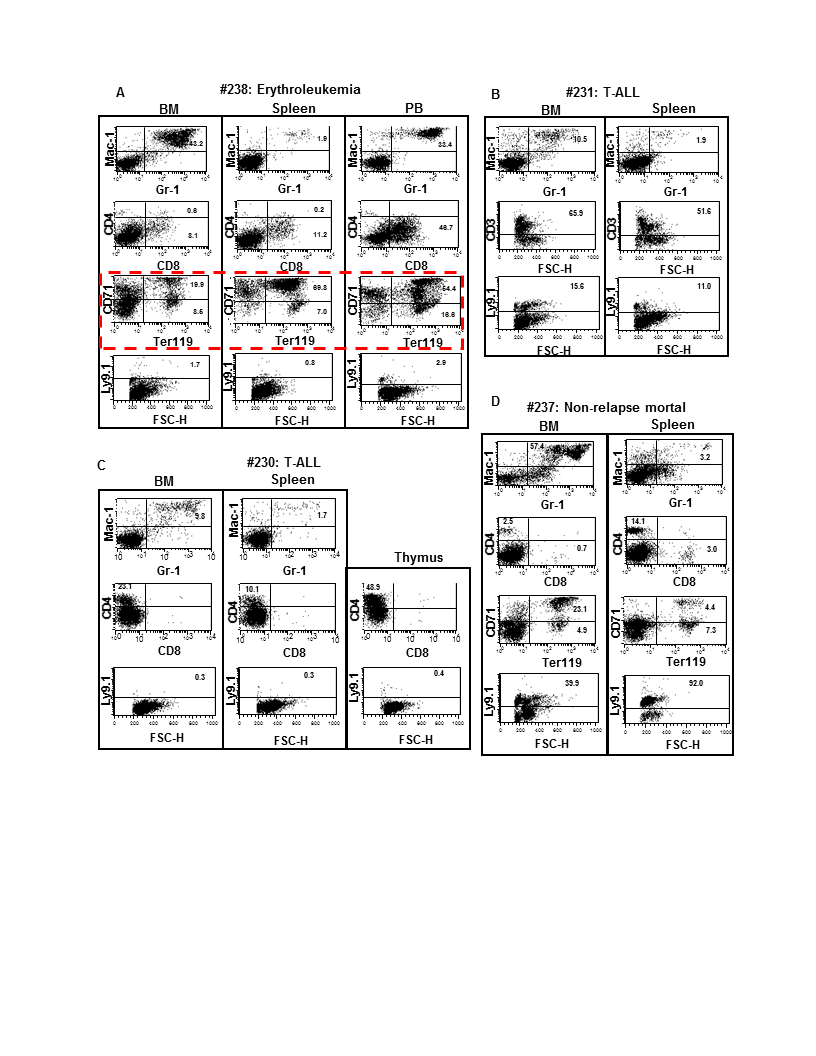

Supplement: S2 Fig — (A) Erythroleukemia, Note increased erythroblast (CD71+/Ter119+) cells in spleen and peripheral blood (PB). (B) and (C) T-ALL characterized by T cell infiltration of BM and predominance of host (Ly9.1 negative) cells. (D) this recipient showed normal immunophenotypic characteristics and predominance of donor cells in spleen. No sign of relapse. (TIF) [file pone.0185219.s002.TIF]

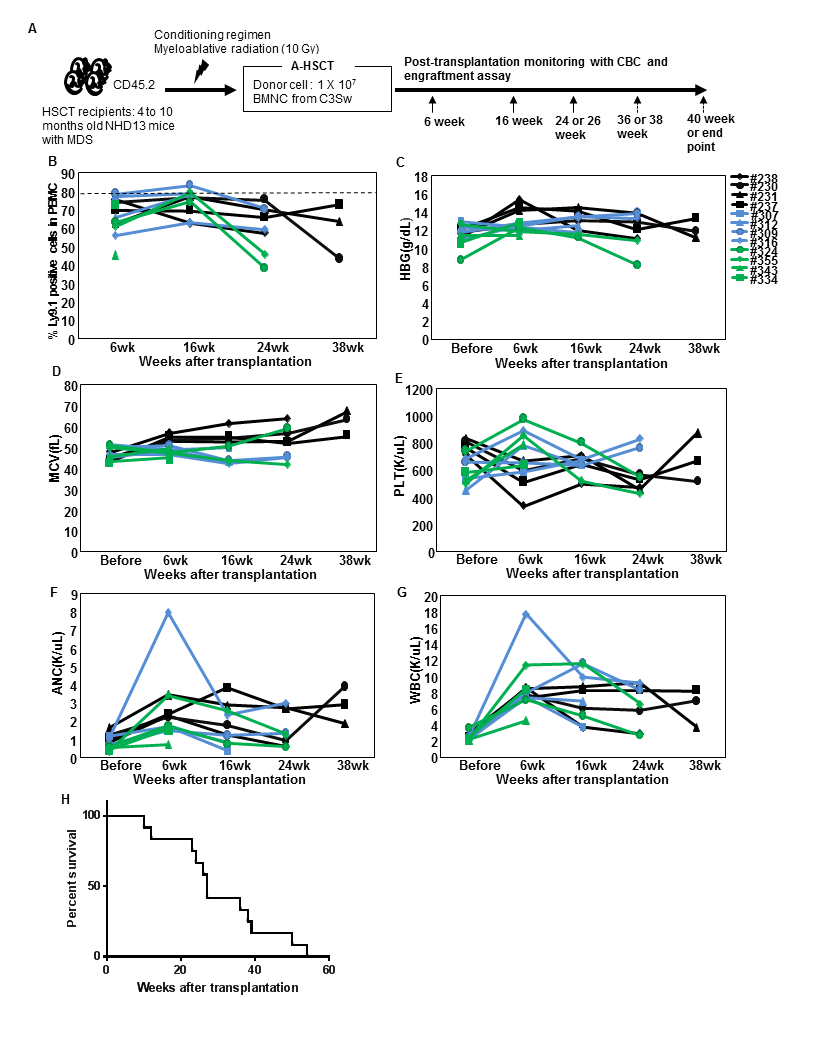

Supplement: S3 Fig — (A) Schematic illustration of A-HSCT experiment. (B) Engraftment of donor cells were evaluated using an anti-Ly9.1 antibody. The dotted line indicates Ly9.1 positivity (79.9 ± 2.5%) in the peripheral blood of 5 healthy C3H.sw donor mice, as a reference for full donor chimerism. (C) HGB, hemoglobin. (D) MCV, Mean corpuscular volume. (E) PLT, platelet count. (F) ANC, absolute neutrophil count. (G) WBC, white blood cell count. (H) Survival curve (n = 12). Three independent A-HSCT experiment cohorts are represented with three different color lines (black, blue and green). Each symbol indicates individual HSCT recipients. (TIF) [file pone.0185219.s003.TIF]

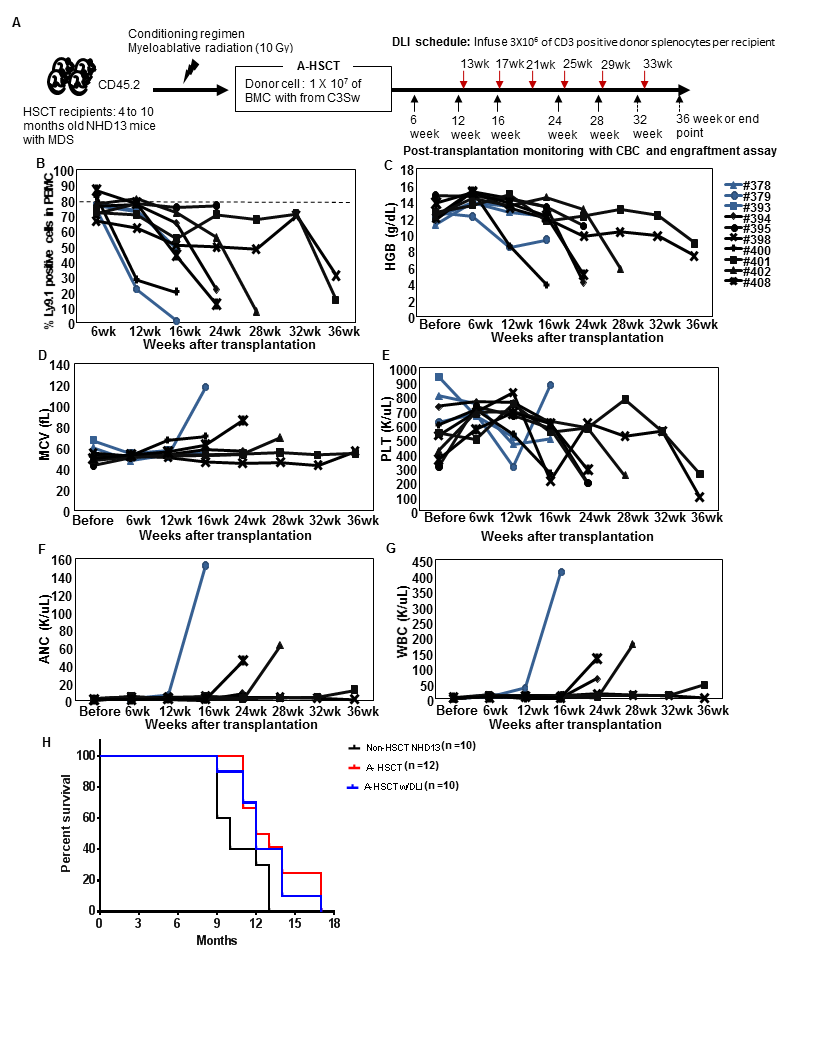

Supplement: S4 Fig — (A) Schematic illustration of the experiment. DLI started at post-transplant week 13 and donor lymphocytes were injected every 4 weeks until the endpoint of the experiment. (B) Engraftment of donor cells were evaluated using an anti-Ly9.1 antibody as described in S3 Fig. (C) HGB, hemoglobin. (D) MCV, Mean corpuscular volume. (E) PLT, platelet count. (F) ANC, absolute neutrophil count. (G) WBC, white blood cell count. (H) Comparing survival curves from A-HSCT and A-HSCT with DLI recipient group. Two independent A-HSCT experiment cohorts are represented by different color lines (black and blue). (TIF) [file pone.0185219.s004.TIF]

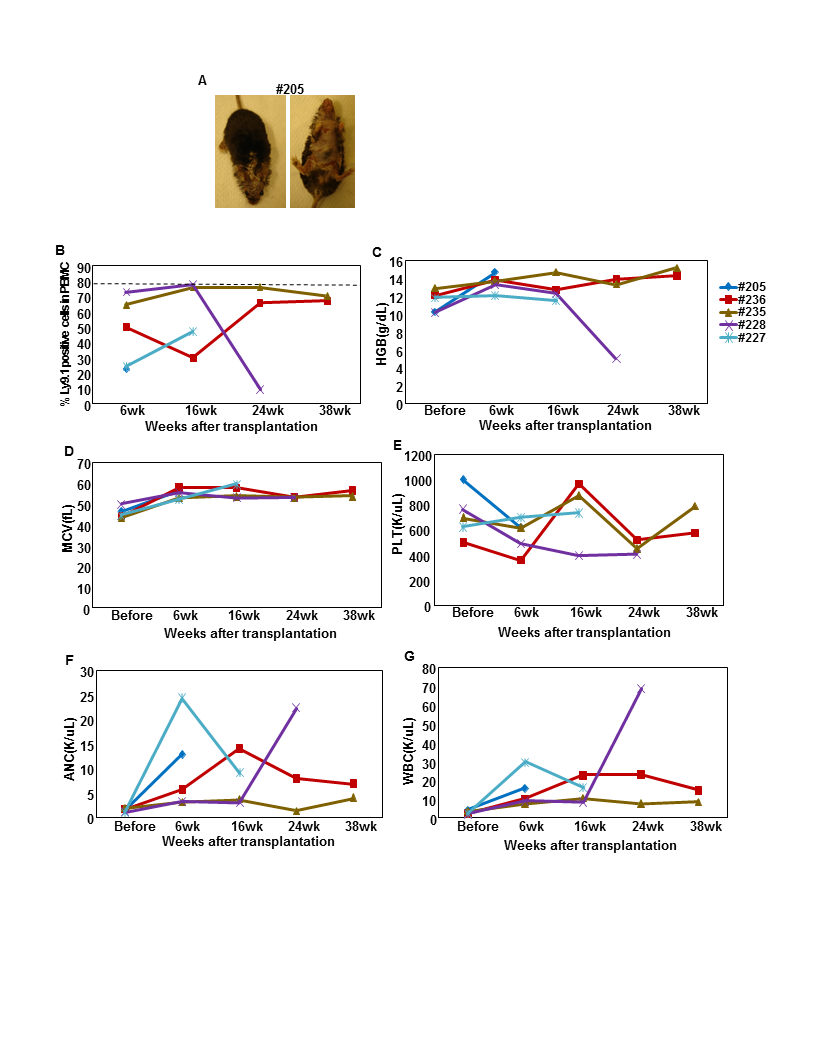

Supplement: S5 Fig — Myeloablative recipient MDS mice were transplanted with allogeneic donor BM along with allogeneic donor splenocytes to induce GVHD. (A) Representative photos of recipients that developed GVHD, # indicates the recipient animal’s identification number. (B) Engraftment of donor cells were evaluated using an anti-Ly9.1 antibody as described in S3 Fig. (C) HGB, hemoglobin. (D) MCV, Mean corpuscular volume. (E) PLT, platelet count. (F) ANC, absolute neutrophil count. (G) WBC, white blood cell count. (TIF) [file pone.0185219.s005.TIF]

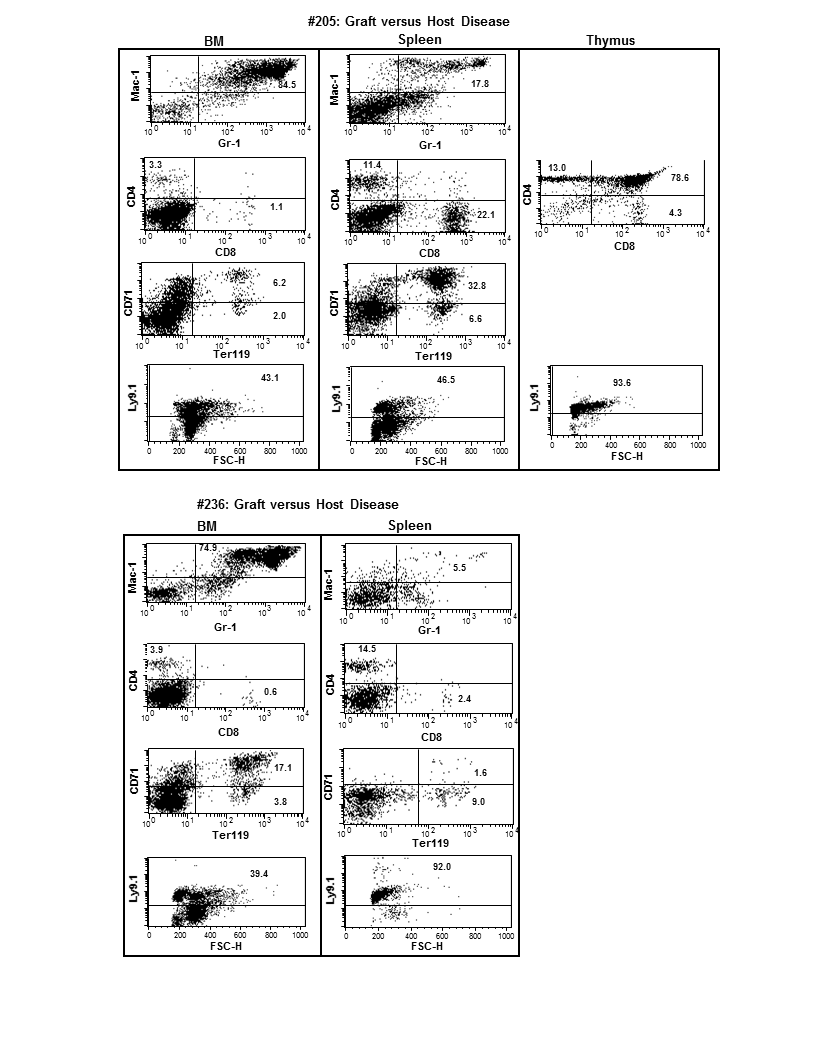

Supplement: S6 Fig — Two recipients that developed GVHD show high level of donor cell engraftment and normal FACS profiles in terms of lymphoid and myeloid cells in hematopoietic tissues. (TIF) [file pone.0185219.s006.TIF]

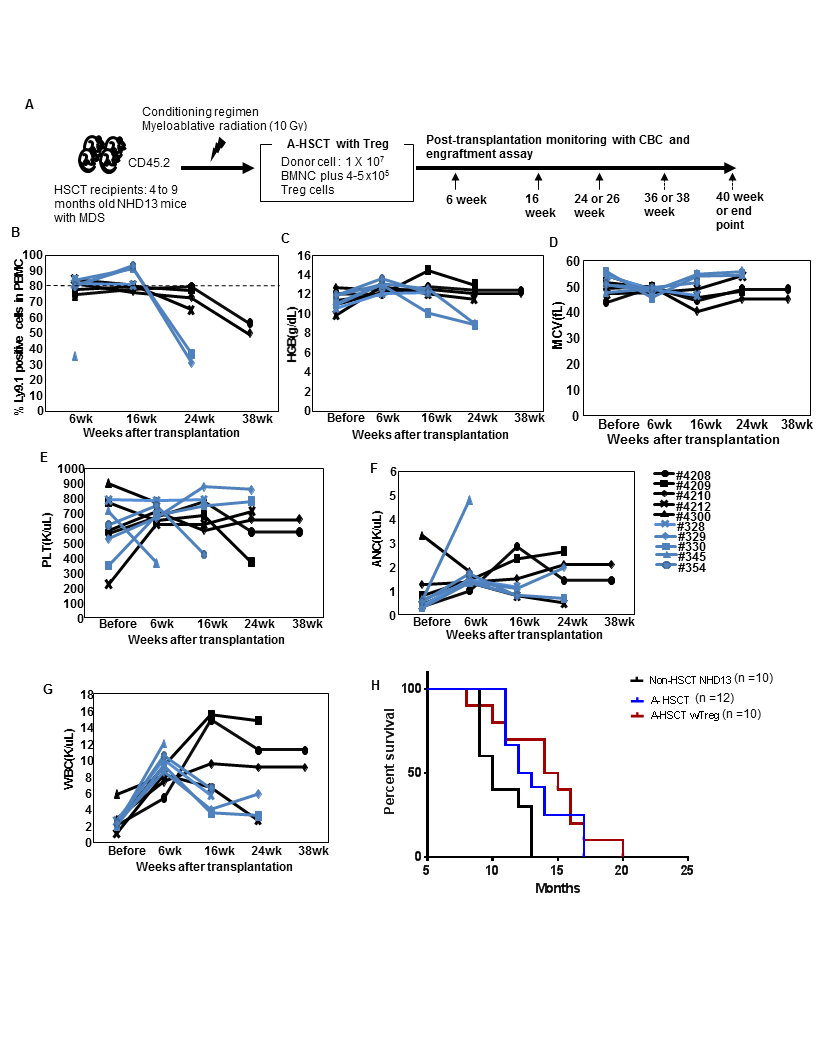

Supplement: S7 Fig — (A) Schematic illustration of A-HSCT plus Treg. CD4+CD25+ T cells were isolated from donor spleen using magnetic cell sorting system (MACS). 4 to 5 X 105 Treg cells were transplanted to lethally irradiated MDS recipients along with 1X107 of whole BM cells. (B) Engraftment of donor cells were evaluated using an anti-Ly9.1 antibody as in S3 Fig. (C) HGB, hemoglobin. (D) MCV, Mean corpuscular volume. (E) PLT, platelet count. (F) ANC, absolute neutrophil count. (G) WBC, white blood cell count. (H) Comparison of survival curves from A-HSCT and A-HSCT with Treg. (TIF) [file pone.0185219.s007.TIF]
