# Supplementary material for: Myeloablative hematopoietic stem cell transplantation improves survival but is not curative in a pre-clinical model of myelodysplastic syndrome
Source: PLoS One. 2017 Sep 27;12(9):e0185219. doi: 10.1371/journal.pone.0185219 (PMC5617185; doi:10.1371/journal.pone.0185219)
Supplement: S1 Table — (DOC) [file pone.0185219.s008.doc]

| Mouse ID | HGB (g/dL) | MCV (fL) | PLT (K/uL) | WBC (K/uL) | ANC (K/uL) | Age (Month) |
| --- | --- | --- | --- | --- | --- | --- |
| WT#1836 | 15.2 | 48.3 | 865 | 7.36 | 4.69 | 8 |
| WT#1840 | 15.7 | 49.9 | 538 | 7.22 | 2.5 | 8 |
| WT#1850 | 17.7 | 47.7 | 745 | 12.94 | 6.91 | 8 |
| WT#1858 | 17.4 | 48.1 | 746 | 8.80 | 2.25 | 8 |
| WT#1892 | 17.1 | 55.2 | 537 | 8.86 | 3.09 | 8 |
| WT#1878 | 15.3 | 53.0 | 756 | 8.84 | 3.81 | 8 |
| WT#1905 | 14.0 | 57.5 | 664 | 10.18 | 3.31 | 8 |
| WT#1906 | 15.8 | 52.3 | 587 | 6.22 | 2.02 | 8 |
| WT#1932 | 14.0 | 51.1 | 607 | 3.34 | 1.37 | 8 |
| WT#1939 | 14.7 | 52.1 | 1101 | 11.36 | 3.66 | 8 |
| WT#1954 | 13.9 | 57.2 | 1003 | 4.58 | 1.01 | 8 |
| WT#1955 | 11.4 | 50.2 | 849 | 2.76 | 1.22 | 8 |
| WT#1968 | 14.6 | 47.9 | 835 | 2.42 | 0.59 | 8 |
| WT#1969 | 14.1 | 50.2 | 1069 | 4.78 | 1.38 | 8 |
| Mean | 15.1 | 51.5 | 779 | 7.12 | 2.70 |  |
| sem | 0.4 | 0.9 | 50 | 0.87 | 0.46 |  |

**S1 Table. Complete Blood Counts (CBC) of C57BL/6 wild type mice**

Wild type CBC data from Gough *et al*. PLos One. 2012;7:e36876.
