# Supplementary material for: Myeloablative hematopoietic stem cell transplantation improves survival but is not curative in a pre-clinical model of myelodysplastic syndrome
Source: PLoS One. 2017 Sep 27;12(9):e0185219. doi: 10.1371/journal.pone.0185219 (PMC5617185; doi:10.1371/journal.pone.0185219)
Supplement: S2 Table — (DOC) [file pone.0185219.s009.doc]

| **S2 Table. CBC of NHD13 transgenic recipients before HSCT** | | | | | | | |
| --- | --- | --- | --- | --- | --- | --- | --- |
| Mouse ID | HGB (g/dL) | MCV (fL) | PLT (K/uL) | WBC (K/uL) | ANC (K/uL) | Age (Month) | Type of HSCT |
| #86 | 13.2 | 55.3 | 606 | 3.48 | 1.77 | 5 |  |
| #87 | 11.5 | 49.9 | 294 | 3.34 | 1.86 | 5 |  |
| #89 | 13.6 | 50.1 | 633 | 3.42 | 2.18 | 5 |  |
| #81 | 11.1 | 58.0 | 424 | 2.82 | 1.32 | 7 |  |
| #84 | 12.1 | 51.1 | 558 | 5.62 | 2.79 | 5 | Syngeneic |
| #90 | 14.2 | 51.1 | 476 | 3.02 | 1.42 | 5 |  |
| #169 | 11.2 | 53.2 | 551 | 4.98 | 2.59 | 7 |  |
| #177 | 12.2 | 53.1 | 889 | 3.30 | 1.55 | 6 |  |
| #178 | 13.2 | 46.7 | 605 | 1.88 | 0.88 | 6 |  |
| #179 | 14.4 | 47.1 | 621 | 2.96 | 1.38 | 6 |  |
| #238 | 11.1 | 48.0 | 718 | 2.30 | 0.86 | 4 |  |
| #231 | 11.4 | 44.5 | 832 | 2.92 | 1.66 | 4 |  |
| #230 | 11.8 | 45.3 | 814 | 2.12 | 1.25 | 4 |  |
| #237 | 12.1 | 43.3 | 764 | 2.56 | 0.97 | 4 | Allogeneic |
| #307 | 13.0 | 45.6 | 534 | 2.44 | 1.21 | 5 |  |
| #309 | 11.8 | 50.3 | 655 | 2.56 | 0.72 | 5 |  |
| #312 | 12.3 | 51.4 | 451 | 2.14 | 0.68 | 5 |  |
| #316 | 11.0 | 46.7 | 683 | 2.42 | 1.10 | 5 |  |
| #324 | 8.7 | 50.9 | 740 | 3.64 | 0.59 | 10 |  |
| #343 | 11.4 | 45.7 | 528 | 2.12 | 0.56 | 8 |  |
| #355 | 12.7 | 50.2 | 498 | 2.14 | 0.38 | 6 |  |
| #334 | 10.7 | 43.5 | 580 | 2.40 | 0.44 | 9 |  |
| #205 | 10.3 | 46.5 | 997 | 4.42 | 1.50 | 9 |  |
| #236 | 12.1 | 44.1 | 501 | 2.62 | 1.68 | 4 | Allogeneic |
| #235 | 12.9 | 43.4 | 691 | 3.66 | 1.83 | 4 | BM |
| #228 | 10.2 | 50.0 | 761 | 2.26 | 1.09 | 4 | with splenocyte |
| #227 | 11.8 | 44.6 | 623 | 2.44 | 1.04 | 4 |  |
| #4208 | 12.1 | 43.6 | 565 | 2.02 | 0.32 | 6 |  |
| #4209 | 11.4 | 51.1 | 772 | 2.24 | 0.78 | 6 |  |
| #4210 | 12.7 | 48.9 | 585 | 2.86 | 1.25 | 6 |  |
| #4212 | 9.9 | 46.9 | 224 | 1.06 | 0.38 | 6 | Allogeneic |
| #4300 | 11.0 | 53.7 | 899 | 5.88 | 3.30 | 6 | BM with Treg |
| #328 | 10.6 | 54.0 | 788 | 2.62 | 0.37 | 9 |  |
| #329 | 11.1 | 50.4 | 530 | 2.28 | 0.60 | 9 |  |
| #330 | 11.0 | 55.5 | 350 | 2.02 | 0.28 | 9 |  |
| #345 | 11.9 | 47.3 | 715 | 2.06 | 0.61 | 8 |  |
| #346 | 10.1 | 49.5 | 760 | 3.34 | 0.93 | 8 |  |
| #354 | 11.9 | 50.4 | 621 | 2.38 | 0.55 | 6 |  |
| #378 | 11.2 | 59.8 | 804 | 2.90 | 0.75 | 10 |  |
| #379 | 12.7 | 48.6 | 621 | 3.88 | 1.51 | 10 |  |
| #388 | 13.1 | 52.2 | 604 | 2.30 | 1.15 | 8 |  |
| #390 | 11.4 | 59.9 | 613 | 4.40 | 1.67 | 8 | Allogeneic |
| #393 | 12.0 | 66.6 | 935 | 2.32 | 0.68 | 8 | DLI |
| #394 | 11.7 | 49.8 | 728 | 2.48 | 1.26 | 6 |  |
| #395 | 14.7 | 42.7 | 310 | 2.86 | 1.34 | 6 |  |
| #398 | 11.9 | 54.8 | 527 | 2.98 | 0.41 | 6 |  |
| #400 | 12.1 | 51.3 | 601 | 2.66 | 1.26 | 5 |  |
| #401 | 12.7 | 49.6 | 545 | 3.10 | 1.55 | 5 |  |
| #402 | 12.3 | 47.9 | 420 | 2.82 | 1.30 | 5 |  |
| #407 | 11.8 | 52.1 | 655 | 2.58 | 0.70 | 5 |  |
| #408 | 13.8 | 49.5 | 362 | 3.16 | 1.13 | 5 |  |
| Mean | 11.9 | 49.9 | 618.8 | 2.9 | 1.2 | 6 |  |
| sem | 0.2 | 0.7 | 23.3 | 0.1 | 0.1 | 0.3 |  |
