# Supplementary material for: Myeloablative hematopoietic stem cell transplantation improves survival but is not curative in a pre-clinical model of myelodysplastic syndrome
Source: PLoS One. 2017 Sep 27;12(9):e0185219. doi: 10.1371/journal.pone.0185219 (PMC5617185; doi:10.1371/journal.pone.0185219)
Supplement: S3 Table — (DOC) [file pone.0185219.s010.doc]

**S3 Table. CBC following HSCT of MDS mice using Non-myeloablative (6.5 Gy) or Myeloablative (10 Gy)TBI as conditioning regimen.**

|  | **Post-transplantation 6 week** | | | | | | | **Post-transplantation 16 week** | | | | | | |
| --- | --- | --- | --- | --- | --- | --- | --- | --- | --- | --- | --- | --- | --- | --- |
|  | **Non-HSCT**  **NHD13**  **(n=17)** | **Non-myeloablative** | | | **Myeloablative** | | | **Non-HSCT**  **NHD13**  **(n=13)** | **Non-myeloablative** | | | **Myeloablative** | | |
|  |  | **#86** | **#87** | **#89** | **#81** | **#84** | **#90** |  | **#86** | **#87** | **#89** | **#81** | **#84** | **#90** |
| **WBC (K/uL)** | **3.37±0.8** | **2.58** | **8.36** | **5.86** | **3.98** | **7.04** | **6.00** | **2.43±0.4** | **2.80** | **5.44** | **4.22** | **4.92** | **11.4** | **11.02** |
| **ANC (K/uL)** | **1.91±0.5** | **1.45** | **2.75** | **1.81** | **1.59** | **2.90** | **1.81** | **1.41±0.1** | **1.43** | **1.83** | **1.53** | **1.33** | **2.78** | **2.46** |
| **HGB (g/dL)** | **11.4±0.8** | **13.4** | **14.4** | **14.4** | **13.9** | **13.7** | **13.8** | **10.4±0.6** | **11.8** | **11.2** | **12.5** | **11.4** | **13.6** | **12.5** |
| **MCV (fL)** | **62.0±1.8** | **54.8** | **53.2** | **52.4** | **51.9** | **50.4** | **50.6** | **62.9±2.5** | **54.2** | **57.3** | **51.9** | **57.3** | **47.1** | **43.7** |
| **PLT (K/uL)** | **716±68** | **637** | **453** | **624** | **511** | **912** | **275** | **753±68** | **844** | **263** | **748** | **836** | **855** | **856** |

Non-HSCT NHD13 refers to mean ± standard error of mean age-matched NHD13 mice with MDS from our prior report.
