# Supplementary material for: Myeloablative hematopoietic stem cell transplantation improves survival but is not curative in a pre-clinical model of myelodysplastic syndrome
Source: PLoS One. 2017 Sep 27;12(9):e0185219. doi: 10.1371/journal.pone.0185219 (PMC5617185; doi:10.1371/journal.pone.0185219)
Supplement: S4 Table — (DOC) [file pone.0185219.s011.doc]

**S4 Table. Clinical outcome of allogeneic HSCT with donor lymphocyte infusion (DLI)**

| Type of HSCT | Recipient ID | Follow-up (*weeks) | CBC Acquisition (*week) | WBC (K/uL) | ANC (K/uL) | PLT (K/uL) | HGB (g/dL) | MCV (fL) | Cause of Death | Diagnosis |
| --- | --- | --- | --- | --- | --- | --- | --- | --- | --- | --- |
| Allogenic with DLI | #378 | 31 | 31 | 2.61 | 0.32 | 76 | 11.4 | 53.2 | **Relapse** | **MDS** |
|  | #379 | 16 | 16 | 411.2 | 151.9 | 881 | 9.3 | 117 | **Relapse** | **AML** |
|  | #393 | 16 | 12 | 5.58 | 1.03 | 520 | 13.4 | 57.2 | **Relapse** | T-ALL^a^ |
|  | #394 | 25 | 24 | 65.24 | 7.74 | 197 | 4.1 | 56.2 | **Relapse** | Leukemia^b^ |
|  | #395 | 27 | 27 | 278.0 | 222.7 | 171 | 4.8 | 50.8 | **Relapse** | **AML** |
|  | #398 | 37 | 36 | 3.26 | 0.72 | 103 | 7.4 | 56.0 | **Relapse** | **MDS** |
|  | #400 | 18 | 16 | 1.44 | 0.41 | 262 | 3.8 | 70.3 | **Relapse** | **MDS** |
|  | #401 | 40 | 40 | 77.66 | 25.51 | 292 | 5.7 | 87.3 | **Relapse** | **AML** |
|  | #402 | 30 | 28 | 178.2 | 61.78 | 250 | 5.8 | 69.7 | **Relapse** | **AML** |
|  | #407 | 20 | 16 | 41.5 | 23.96 | 368 | 9.9 | 71.7 | **Relapse** | **AML** |
|  | #408 | 26 | 24 | 133.1 | 45.11 | 290 | 5.1 | 85.8 | **Relapse** | **AML** |

*, Week after transplantation

^a^ #393 recipient with thymoma on necropsy after being found dead

^b^ “Leukemia” indicates leukemia not otherwise specified (NOS) for mice found dead with hepatosplenomegaly at necropsy
