# Supplementary material for: Myeloablative hematopoietic stem cell transplantation improves survival but is not curative in a pre-clinical model of myelodysplastic syndrome
Source: PLoS One. 2017 Sep 27;12(9):e0185219. doi: 10.1371/journal.pone.0185219 (PMC5617185; doi:10.1371/journal.pone.0185219)
Supplement: S5 Table — (DOC) [file pone.0185219.s012.doc]

**S5 Table. Clinical outcome of allogeneic HSCT with regulatory T cells (Treg)**

| Type of HSCT | Recipient ID | Follow-up (*weeks) | CBC Acquisition (*week) | WBC (K/uL) | ANC (K/uL) | PLT (K/uL) | HGB (g/dL) | MCV (fL) | Cause of Death | Diagnosis |
| --- | --- | --- | --- | --- | --- | --- | --- | --- | --- | --- |
| Allogenic with Treg | #4208 | 45 | 38 | 11.22 | 2.1 | 689 | 12.4 | 48.7 | **Relapse** | Leukemia (splenomegaly, pleural effusion) |
|  | #4209 | 33 | 33 | 137 | 6.19 | 69 | 6.3 | 47.9 | **Relapse** | **AML** (Monocyte 66.8 K/uL) |
|  | #4210 | 46 | 38 | 9.16 | 2.1 | 766 | 12.1 | 44.8 | **Relapse** | **MDS** (no sign of leukemia, pleural effusion) |
|  | #4212 | 36 | 24 | 2.76 | 0.48 | 712 | 11.5 | 54.0 | **Relapse** | **MDS** |
|  | #4300 | 10 | 6 | 8.16 | 1.74 | 774 | 12.3 | 48.8 | unknown | Non-relapse mortal |
|  | #328 | 22 | 16 | 5.78 | 1.14 | 789 | 12.3 | 46.3 | **Relapse** | Leukemia (hepatosplenomegaly) |
|  | #329 | 28 | 28 | 16.9 | 1.62 | 103 | 3.3 | 65.2 | **Relapse** | **AML** (splenomegaly, 32.3% Blasts in BMC) |
|  | #330 | 43 | 43 | 30.5 | 25.13 | 47 | 3.1 | 60.3 | **Relapse** | **AML** |
|  | #345 | 10 | 10 | 1.2 | 0.08 | 89 | 2.7 | 55.1 | **Relapse** | **MDS** |
|  | #346 | 6 | 6 | 144.5 | 28.77 | 167 | 5.7 | 58.3 | **Relapse** | **T-ALL** |
|  | #354 | 24 | 24 | 7.69 | 0.66 | 41 | 3 | 55.2 | **Relapse** | **MDS** |

*, Week after transplantation; “Leukemia” indicate not otherwise specified (NOS), mice found dead with hepatosplenomegaly at necropsy.
